# Improved grazing management may increase soil carbon sequestration in temperate steppe

**DOI:** 10.1038/srep10892

**Published:** 2015-07-03

**Authors:** Wenqing Chen, Ding Huang, Nan Liu, Yingjun Zhang, Warwick B. Badgery, Xiaoya Wang, Yue Shen

**Affiliations:** 1 Department of Grassland Science, College of Animal Science and Technology, China Agricultural University, West Road 2 Yuan Ming Yuan, Beijing 100193, P.R. China; 2 New South Wales Department of Primary Industries, Orange Agricultural Institute, Orange, NSW 2800, Australia

## Abstract

Different grazing strategies impact grassland plant production and may also regulate the soil carbon formation. For a site in semiarid temperate steppe, we studied the effect of combinations of rest, high and moderate grazing pressure over three stages of the growing season, on the process involved in soil carbon sequestration. Results show that constant moderate grazing (MMM) exhibited the highest root production and turnover accumulating the most soil carbon. While deferred grazing (RHM and RMH) sequestered less soil carbon compared to MMM, they showed higher standing root mass, maintained a more desirable pasture composition, and had better ability to retain soil N. Constant high grazing pressure (HHH) caused diminished above- and belowground plant production, more soil N losses and an unfavorable microbial environment and had reduced carbon input. Reducing grazing pressure in the last grazing stage (HHM) still had a negative impact on soil carbon. Regression analyses show that adjusting stocking rate to ~5SE/ha with ~40% vegetation utilization rate can get the most carbon accrual. Overall, the soil carbon sequestration in the temperate grassland is affected by the grazing regime that is applied, and grazing can be altered to improve soil carbon sequestration in the temperate steppe.

Soil is the major terrestrial reservoir of carbon, storing more than twice the amount of carbon than the atmosphere^1^ as decomposed plant litter and residue^2^. Any changes in soil carbon storage have the potential to modify the global carbon cycle and influence climate change^3^. Studies have estimated that adopting appropriate land management practices alone might offset about a third of the global annual greenhouse-gas emissions^4^. Grasslands comprise approximately 40% of the earth’s land area and play a critical role in the global carbon cycle^5^. Grazing, as the most geographically expansive grassland use today, can exert significant influence on more than a quarter of the global potential for soil carbon storage^6^. Although grazing converts consumed plants into CO_2_ and other greenhouse gases, multiple studies have shown that grazing can often lower net ecosystem carbon emissions and promote soil carbon storage^7^, especially in dry ecosystems^8^. Thus, facilitating soil carbon sequestration through improved grazing regimes in these regions is an important measure for offsetting greenhouse-gas emissions to mitigate current climate change.

Higher grazing intensity is generally thought to decrease soil carbon in C3-dominated grasslands by reducing CO_2_ fixation from the loss of photosynthetic tissue and reducing belowground carbon inputs through lower root production and higher turnover of root litter^9^,^10^. However, higher grazing intensity can be associated with increased SOC in grasslands dominated by C4 grasses due to a stimulation of fine, shallow roots by grazing^11^,^12^. For mixed C3 and C4 grasslands or pastures, grazing has a positive effect at both light and heavy grazing intensities and a negative effect at moderate intensities^13^. Grazing may alter soil organic matter decomposition through its effect on soil microbes, which is critical to the stabilization of SOC^14^. The grazing regime and intensity influences the biomass and diversity of microbes, which consequently controls soil carbon turnover^15^. Moreover, the fungal:bacterial dominance of a site has been associated with C-sequestration potential. Sites with a greater fungal:bacterial dominance are associated with higher soil carbon levels^14^. However, the positive relationship between fungal dominance and C-sequestration may not be a general phenomenon^16^. Such variable responses of soil carbon to grazing could have multiple environmental causes but could also imply that grazing-effects are highly site-specific and grazers in different regions might be managed differently to help increase soil carbon sequestration.

Traditional grazing management practices in the temperate steppe of China are often focused on supplying feed to livestock without consideration of the persistence of desirable grasses. This is widely thought to be the major cause of grassland degradation and the subsequent decrease in soil carbon pools. Improved grazing management regimes have been considered as an important strategy to renew degraded grassland and to promote carbon storage in the soil^3^,^17^. Therefore, an approach that promotes sustainable use of grasslands was launched across this region. The new management techniques include reduced stocking rates or tactical rests, and adjusting stocking rates at different times of the grazing season. Soil organic carbon formation in these grasslands is regulated by the grazing regimes that are applied, but whether and how these grazing regimes affect plant production and soil carbon inputs of the ecosystem remains unclear.

This study investigated livestock production systems to determine the effect of different seasonal grazing pressures on the process involved in soil carbon sequestration. Combinations of rest, high and moderate grazing pressure were implemented over three grazing stages. Our objective was to address: 1) how soil carbon responds to varying seasonal grazing pressures, 2) what major mechanisms mediate soil carbon input under these grazing regimes, and 3) what optimal grazing pressure is suitable for soil carbon sequestration in the temperate steppe.

## Results

### Grazing intensity and vegetation utilization rate

The standardized stocking rate [50 kg Sheep Equivalent (SE)] for each regime (treatment), across all grazing years and stages is shown in supplementary Fig. S2a. The average stocking rate for the grazing season varied substantially between regimes and was 7.52 SE/ha for HHH, 6.51 for HHM, 5.59 for MMM, 3.45 for RHM and 3.41 for RMH (Supplementary Fig. S2b).The vegetation utilization rate (UR) for each stage ranged between 0 and 0.7 and the averaged UR across stages for each regime was in the range of 0.3–0.64 (Supplementary Fig. S3a,b).

### Grazing effects on plant above- and belowground compartments

**Figure 1:**
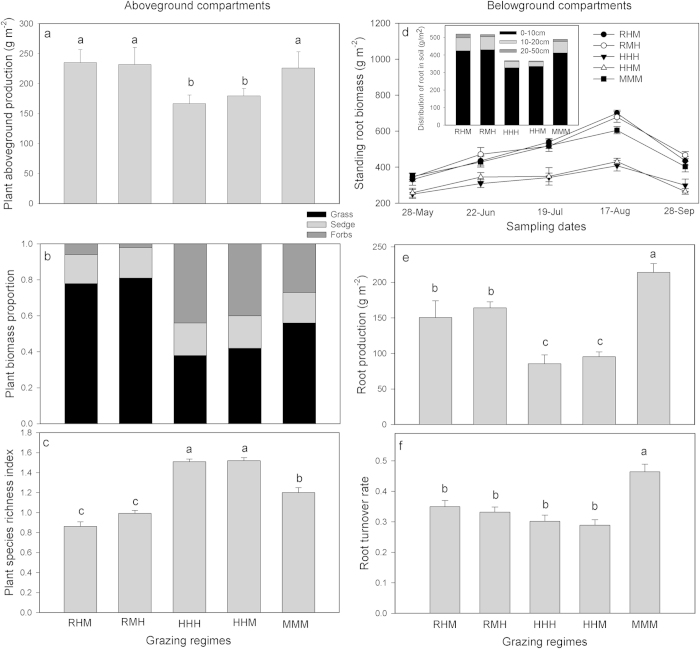
Effects of different grazing regimes on plant aboveground (**a–c**) and belowground compartments (**d–f**) measured in 2013. Significant differences (P < 0.05) between regimes are denoted with different lowercase letters. Data are presented as means ± SE.

**Figure 2:**
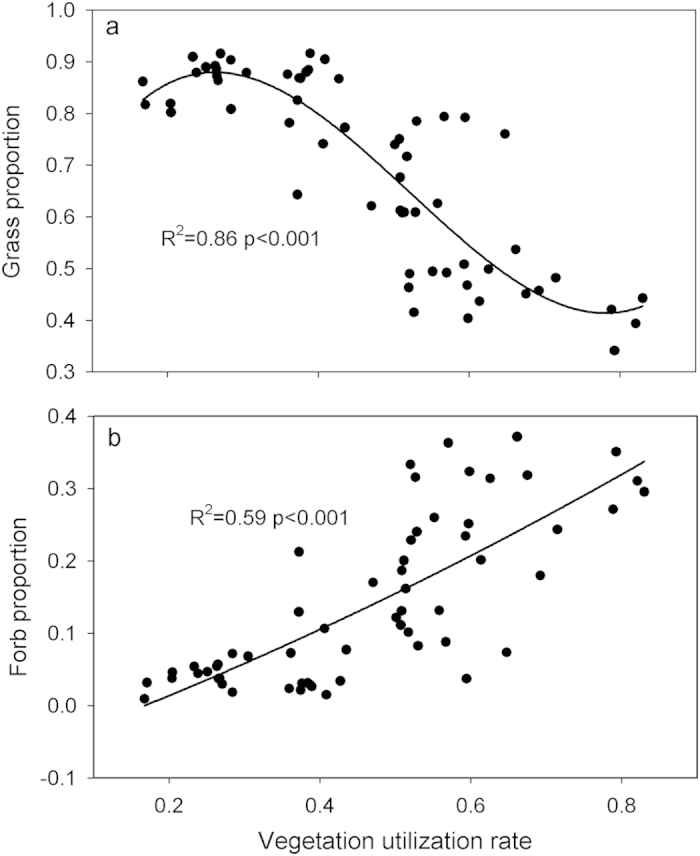
The relationship between vegetation utilization rate (UR) and grass (**a**) or forb proportion (**b**) in the temperate steppe.

Plant aboveground production was significantly influenced by grazing regimes (F_4,10_ = 46.8, p < 0.05; Fig.1a). The aboveground plant production in the two deferred grazing regimes was similar to that in MMM (Duncan’s multiple-range tests, p > 0.05) but significantly higher than HHH and HHM (p < 0.05; Fig. 1a). Grazing regimes led to strong shifts in plant species richness and vegetation composition as measured in mid-August 2013 (Fig. 1b,c). C3 grasses represented approximately 78–81% of the aboveground biomass in RHM and RMH, and approximately 58% in MMM. The proportion of C3 grasses was only 38–42% in HHH and HHM. The proportion of C3 grasses decreased and the proportion of forbs increased as stocking rates increased (Fig. 2a,b).

**Figure 3:**
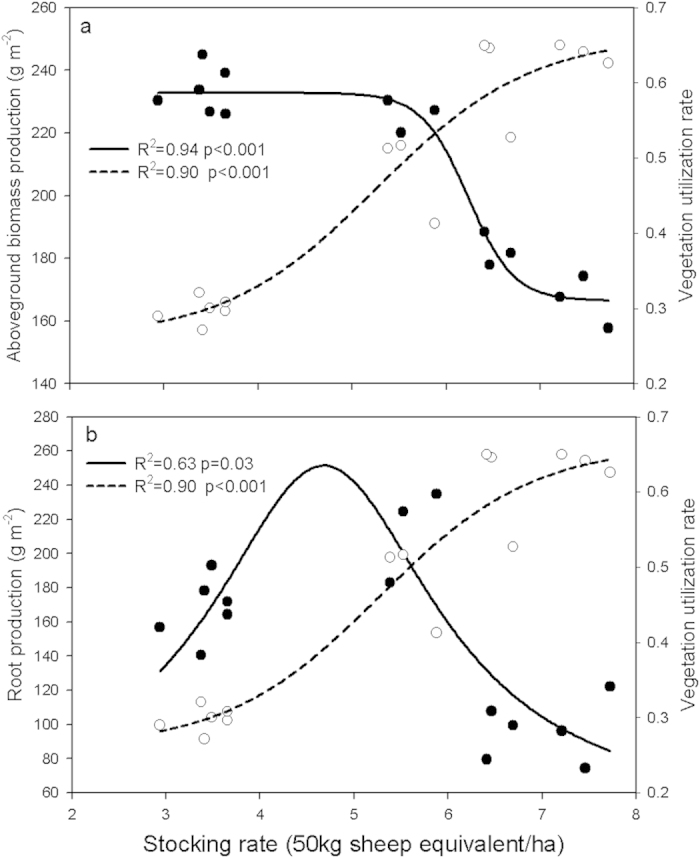
The relationship between stocking rates and aboveground biomass production (**a**, solid symbols), belowground root production (**b**, solid symbols) and vegetation utilization rate (UR) (**a,b**, open symbols, each symbol was the average value for all grazing years). Nonlinear relationship: stocking rates with aboveground biomass production, solid line y = 168.56 + 67.3/(1 + e^(−(x−6.27)/−0.15)^); stocking rates with belowground root production, solid line y = 45.92 + 205.53/(1 + ((x−4.68)/1.47)^2^); stocking rates with UR, short dashed line y = 0.26 + 0.41/(1 + e^(−(x−5.3)/0.82)^).

Standing root biomass was affected by grazing regimes and also showed marked seasonal variations throughout the grazing periods (p < 0.05; Fig.1d). When averaged across all dates, there was no difference in the standing root biomass between RHM and RMH (Duncan’s multiple-range tests, p > 0.05) but it was significantly higher compared with MMM (p < 0.05; Fig.1d). Also, no difference was observed between HHH and HHM (p > 0.05), which showed the lowest average standing root biomass. The root mass was largely restricted to the top soil zone, with more than 85% root distributing in the 0-10 cm soil layer for each treatment. Grazing exerted a significant influence on belowground root production (F_4,10_ = 16.8, p < 0.05; Fig. 1e) and turnover (F_4,10_ = 5.5, p < 0.05; Fig. 1f).The root production and turnover of MMM were both significantly higher than other regimes (Duncan’s multiple-range tests, p < 0.05). HHH and HHM showed no difference in root production (p > 0.05), but were significantly lower than the two deferred grazing regimes (p < 0.05; Fig. 1e,f). The vegetation UR showed a consistent increase with increasing stocking rates, whereas the aboveground production was relatively stable at stocking rates below ~5.4 SE/ha with a sharp decline after that (Fig. 3a). The root production had a different response to stocking rate compared to aboveground production, increasing to a peak at approximately ~4.5 SE/ha and then decreased (Fig. 3b).

Grazing regimes caused no change to the percentage of N, lignin, the lignin:N and C:N ratios in the roots (F_4,10_ = 0.49, p = 0.74; F_4,10_ = 1.32, p = 0.33; F_4,10_ = 1.11, p = 0.41; F_4,10_ = 0.96, p = 0.47; Supplementary Fig. S4b). However, the grazing regimes changed all these quality variables in the shoots (F_4,10_ = 394.4, p < 0.05; F_4,10_ = 36.6, p < 0.05; F_4,10_ = 175.9, p < 0.05; F_4,10_ = 33.5, p < 0.05; Supplementary Fig. S4a).

### Grazing effect on the soil carbon and nitrogen contents, and microbial community

**Figure 4:**
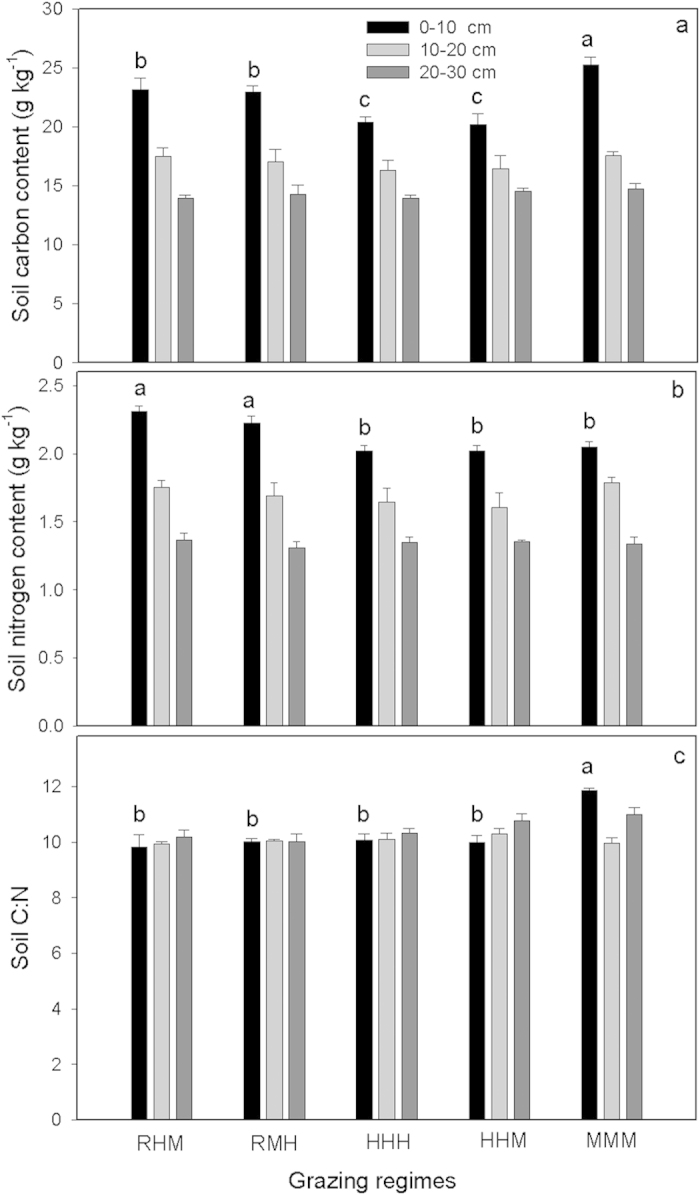
Soil organic carbon (**a**) total nitrogen (**b**) and C:N (**c**) of 0–30 cm soil layers from different grazing regimes. Different lowercase letters indicate significant differences (P < 0.05) in soil organic carbon, total nitrogen and C:N between treatments. Values are mean ± SE.

**Figure 5:**
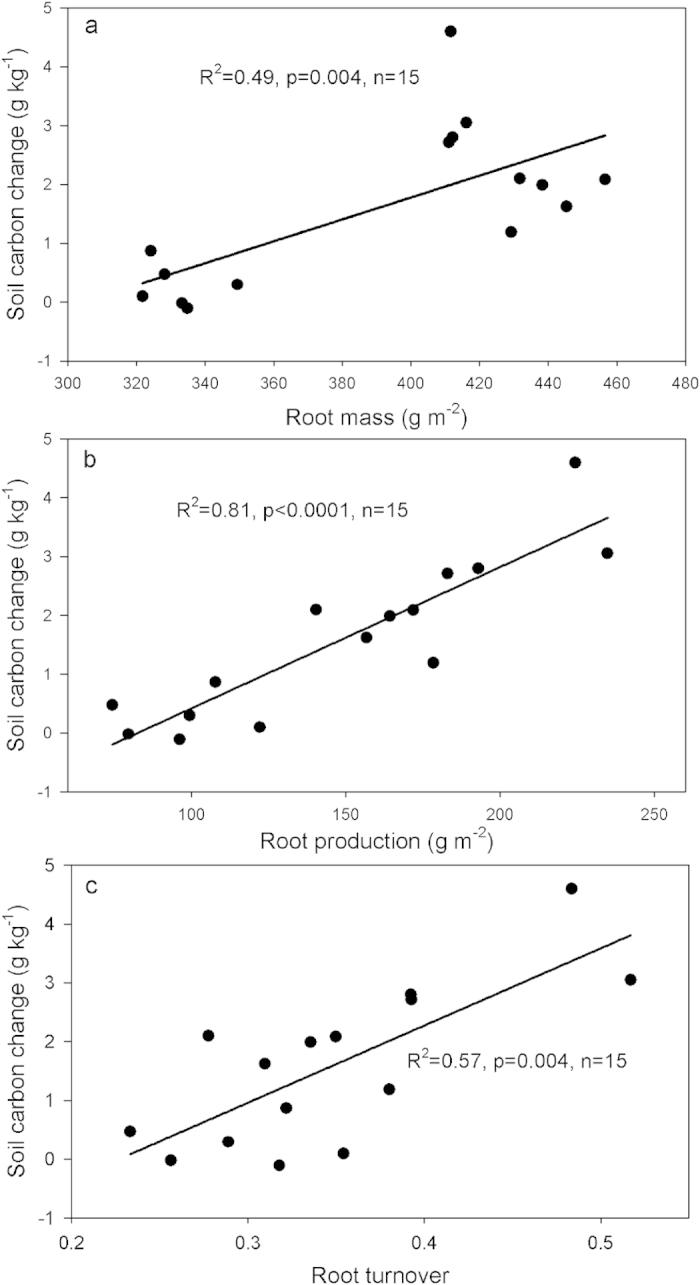
The relationship between soil organic carbon change in the top 10 cm soil with belowground root mass measured in August (**a**) root production (**b**) and root turnover rate (**c**). Linear relationship for (**a**) (**b**) and (**c**).

Soil carbon content was affected by grazing and decreased with sampling depth (p < 0.05; Fig. 4a).There were significant interactions (P < 0.05) between grazing and sampling depth. Soil organic carbon change only occurred in the top 0–10 cm of the soil. The soil carbon content of MMM was significantly higher than other regimes (p < 0.05). RHM and RMH showed no difference in soil carbon (p > 0.05), but were significantly higher than HHH and HHM (p < 0.05). The top soil carbon change was positively correlated with the belowground root mass, root production and root turnover rate (Fig. 5a–c).

**Figure 6:**
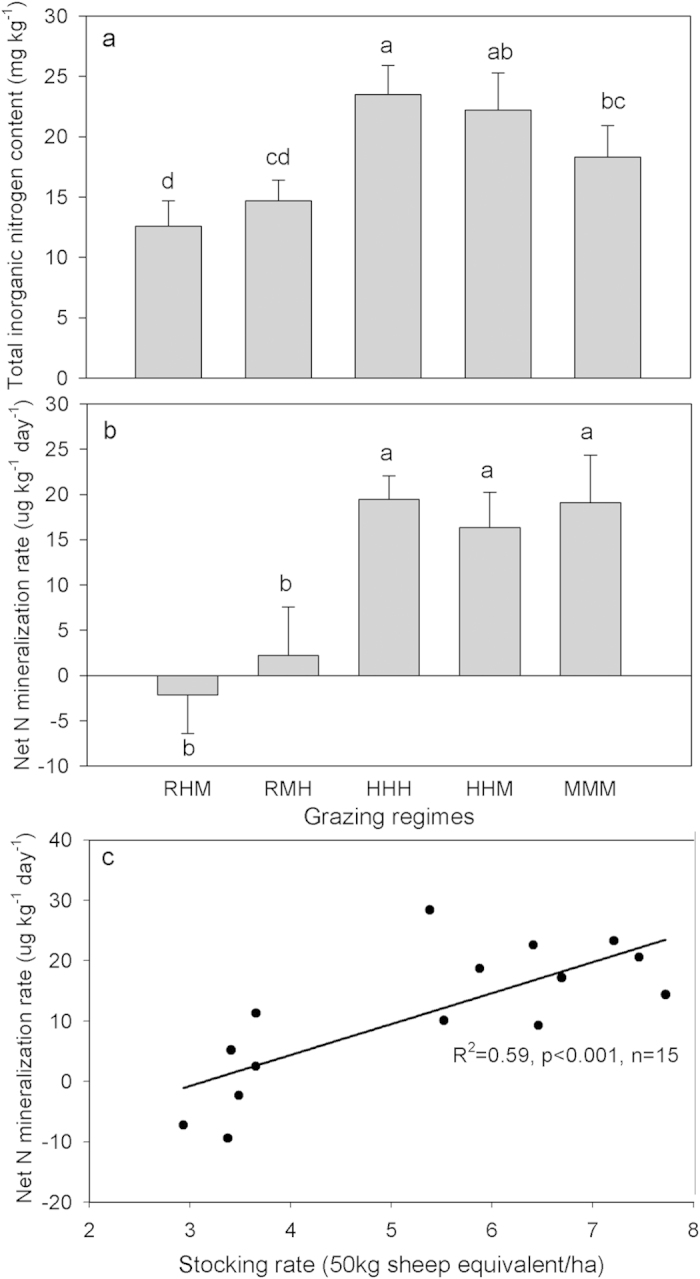
Soil inorganic nitrogen contents (**a**) and net N mineralization rate (**b**) measured in mid-August 2013 in each regime, and the linear relationship between stocking rate with N mineralization rate (**c**). Bar groups with different lowercase letters indicate significant differences (P < 0.05) between regimes for (**a,b**) and data are means ± SE.

Similarly, grazing and soil depth effects (p < 0.05) were also detected for soil total nitrogen, and their interaction effects were significant (p < 0.05; Fig. 4b). The change in total soil nitrogen due to grazing was also observed in the top soil, but with a different pattern of change to soil organic carbon. Grazing regimes significantly affected the soil inorganic nitrogen contents and nitrogen mineralization in the top soil (F_4,10_ = 9.21, p < 0.05; F_4,10_ = 5.42, p < 0.05; Fig. 6a,b). Soil inorganic nitrogen contents showed no difference between HHH, HHM and MMM (Duncan’s multiple-range tests, p > 0.05), but were significantly higher than RHM and RMH (p < 0.05; Fig. 6a). The nitrogen mineralization rates measured during the grazing season were very low, even exhibiting immobilization under deferred grazing regimes. Conversely, the rates were significantly enhanced under HHH, HHM and MMM (p < 0.05; Fig. 6b). The net N mineralization showed a linear increase with increased stocking rate (Fig. 6c).

**Figure 7:**
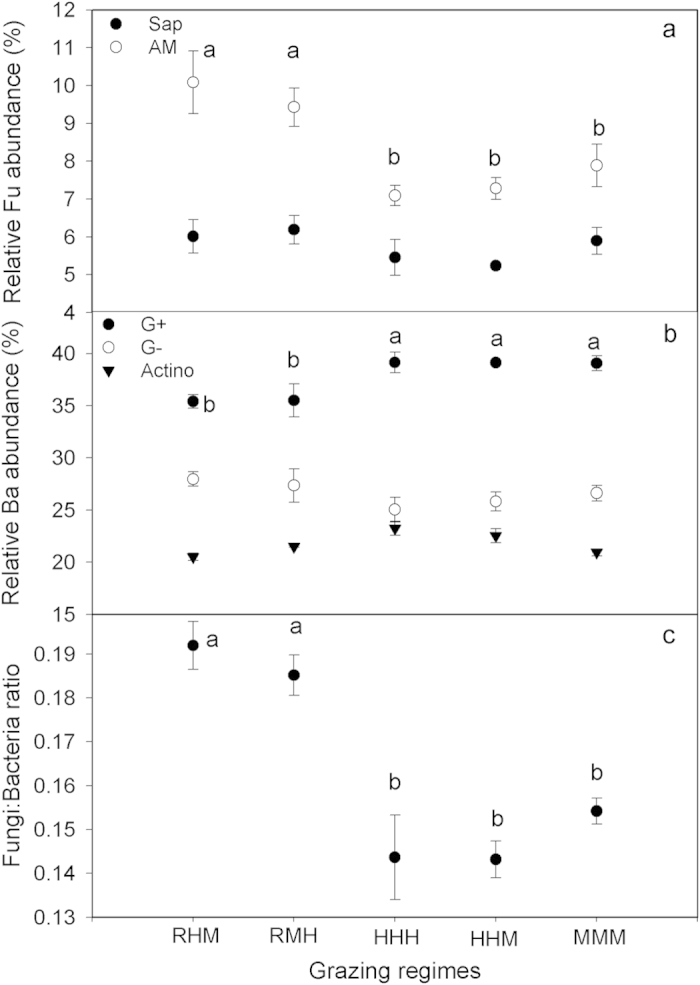
Changes induced by grazing regimes in soil microbial community structure expressed as relative abundance of fungi (**a**) bacteria (**b**) and fungi:bacteria ratio (**c**) from a typical steppe. Solid symbols with different lowercase letters indicate significant differences (P < 0.05) in the abundance of corresponding microbial group. Values are mean ± SE. Sap saprophytic fungi, AM arbuscular mycorrhiza, G( + ) Gram-positive bacteria, G( − ) Gram-negative bacteria, Actino actinomycetes bacteria.

Grazing changed the relative abundance of the microbial functional groups (Fig. 7a–c). The relative abundance of Arbuscular mycorrhiza was significantly lower in HHH, HHM and MMM than in the two deferred grazing regimes (Duncan’s multiple-range tests, p < 0.05; Fig. 7a). The Gram ( + ) bacteria, which had the highest abundance in the microbial community, showed significantly higher abundance levels in HHH and HHM than RMH and RHM (p < 0.05), but exhibited no difference when compared with MMM (p > 0.05; Fig. 7b). The fungi:bacteria ratios (F:B) observed in HHH, HHM and MMM were lower than those in RHM and RMH (p < 0.05; Fig. 7c).

**Figure 8:**
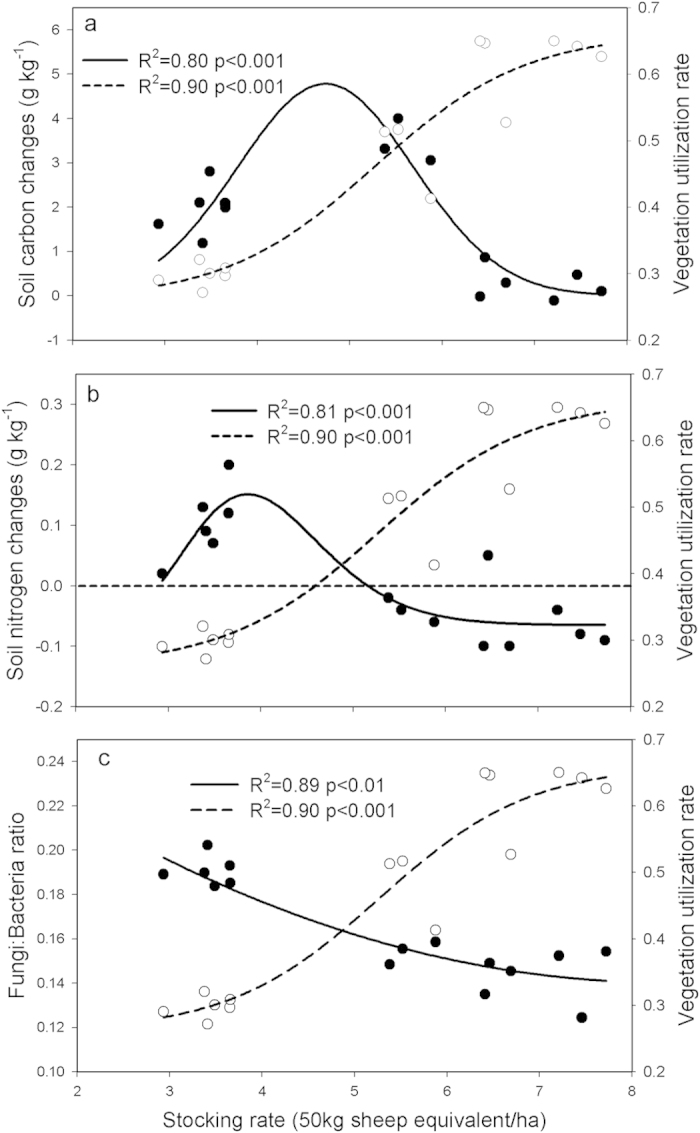
The relationship between stocking rates and soil carbon change (**a**, solid symbols), soil nitrogen change (**b**, solid symbols), fungi:bacteria (F:B) (**c**, solid symbols) and vegetation utilization rate (UR) (**a–c**, open symbols). Nonlinear relationship: carbon change with stocking rates, solid line y = 4.8 × e^(−0.50×((x−4.71)/0.95)2)^; Nitrogen change with stocking rates, solid line y = –0.06 + 0.22 × e^(−0.5×(ln(x/3.92)/0.19)2)^; F:B ratio with stocking rates, solid line y = 0.27−0.03 × x + 0.002 × x^2^.

Further analysis revealed that there is an increase in carbon accumulation, as stocking rate increases, to the biological optimal stocking rate of ~5 SE/ha with ~40% vegetation UR (Fig. 8a). The relationship between nitrogen changes and stocking rates showed a partial decoupling with carbon changes, with the optimal stocking rate at ~4 SE/ha coupled with ~30% vegetation UR (Fig. 8b). The F:B continuously decreased with increased stocking rates (Fig. 8c).

## Discussion

By 2013, after four years of grazing, the grazing regime was responsible for (i) changes in aboveground production; and (ii) variation in vegetation composition in this temperate grassland. The relationship between the proportion of grasses and forbs with stocking rate illustrated that grazing can change aboveground biomass production indirectly through altering vegetation composition and directly with high utilisation reducing plant production. However, grazing induced changes to aboveground production might contribute little to the soil carbon change because much of the aboveground mass consumed and respired by animals would be respired anyway by microbes during degradation and would not be available for soil carbon formation anyway^18^.

Belowground biomass grows in the soil and contributes higher levels of organic matter to soils^19^. The soil carbon change in this study was largely restricted to the top 10 cm soil layer, which has the highest root mass. A recent review, which included 257 published studies, also shows that soil carbon storage can be positively related to changes in belowground root mass^20^. In this study, we found that standing root mass, root production and turnover rate were all significantly influenced by the grazing regime and soil carbon change was positively related to these variables (Fig. 5a–c). This gives further confirmation that soil carbon change is tightly associated with changes in roots, which vary with the management of grazing systems. The lowest root production, biomass and turnover were observed in the HHH and HHM, which decreased the carbon input to soil, resulting in lower soil carbon accumulation. As we found no difference in soil bulk density among treatments in this study, possibly due to the relatively short history of grazing treatments or the freezing and thawing cycles in this area, soil compaction was not associated with the reduction in belowground production under the high grazing pressure. The response of roots under these two regimes may be due to reduced carbon allocation to roots as a result of the high utilization of photosynthetic tissue by herbivores^21^ and the increased proportion of annual forbs, which lack dense fibrous roots. The greatest carbon increase occurred in MMM, which showed the highest root production but not standing root mass. The seemingly paradoxical results between root production and standing root mass in MMM indicated that many of the rapidly produced roots were short lived, such that root standing mass did not accumulate much more standing root mass as expected. Minrhizotron studies that continuously monitored root dynamics found a dramatic burst of root growth after defoliation, but the standing root mass returned to pretreatment levels by the end of growing season due to the rapid senescence^22^. Although we could not continuously monitor the root dynamics using the ingrowth-core method in this study, the large amount of organic matter detritus observed in MMM suggests that the faster root turnover contributed much higher plant carbon into the soil. The observed root responses are likely connected to the rapid production of new foliage after grazing^23^. Although, the root turnover rates were lower in RHM and RMH compared to MMM, the higher standing root mass indicated a high carbon input potential in the long term. Since there were no control plots in this study when grazing treatments initially were imposed, the grazing induced carbon change can be to some extent confounded by the trajectory of carbon recovery at the time the grazing trial was implemented and the effects of a changing environment. However, given the study area we selected is contiguous with the same initial soil conditions and is under similar climate conditions, we therefore assume that these influences are comparable between treatments and the variation in soil carbon is mainly driven by the different grazing regimes that were applied.

Previous studies show that the effects of grazing on plant production is variable, but most have a negative influence on aboveground production^24^,^25^ and positive influence on belowground biomass^26^. Our results, in a temperate steppe environment, show that the aboveground biomass production was resilient to grazing up to a utilization rate of 50% (~5.5 SE/ha), but declined sharply with higher levels of utilization (Fig. 3a). The belowground production seems to be more sensitive than the aboveground production showing a continuous change with stocking rate (Fig. 3b). This may indicate that the root growth is critical to rapidly acquire nutrients to replace lost foliage under moderate grazing. Further regression analysis between carbon change and stocking rate showed that the optimal stocking rate for carbon sequestration is ~5SE/ha (Fig. 8a), which coincided with the stocking rate for the highest root production.

Changes in soil carbon content under grazing should be related to changes in soil nitrogen that, in turn, affect plant production and soil carbon decomposition^18^. Soil total nitrogen contents increased under deferred grazing regimes, but decreased in HHH, HHM and MMM. These differences could be attributed to the variation in soil nitrogen cycling under different forms of grazing management. The rate of nitrogen cycling has been reported to mostly increase under animal grazing due to the conversion of plant tissue into dung and urine^27^ and the alteration of nitrogen content of plant tissue and litter^28^. Here we also found a positive linear relationship between net nitrogen mineralization and stocking rates (Fig. 6c). As suggested before, grazing may accelerate N recycling but also increase N losses. The reduction in soil total nitrogen in HHH, HHM, and MMM might be caused by higher leaching given that seasonal rainfall does coincide with the grazing season or the timing of dung and urine release, but probably more is lost in NH_3_ volatilization. In addition, some nitrogen losses in HHH, HHM and MMM could partly be explained by a redistribution of soil nitrogen into the plant or litter nitrogen since the plant aboveground nitrogen contents were significantly elevated under these grazing regimes (Supplementary Fig. S4a), the redistribution spatially of nitrogen in plots (sheepfold area) where there is a higher stocking rate over the grazing season, and higher export of N as lamb from the systems. However, the high level of soil nitrogen loss in MMM seems to be a surprising finding, because the high carbon input from faster root turnover in this regime did not, as expected, couple with high nitrogen input. The higher root production and turnover in MMM may have increased the flow of plant debris into the soil, resulting in a higher amount of carbon input to soils during decomposition. However, the decoupling of carbon and nitrogen input in our study indirectly reflected the limited nitrogen release during decomposition in MMM. Widespread patterns of litter nitrogen immobilization have been observed during long-term decomposition^29^, which means nitrogen release during litter decomposition is probably much lower. This could be due to strong microbial immobilization of nitrogen during litter decomposition. Litter or plant debris initially has much higher C:N ratios than microbes, and re-immobilization of mineralized nitrogen or N immobilization from exogenous environments is required to meet the stoichiometric balance of microbes^29^,^30^. Besides, there are two other possibilities explaining limited nitrogen release: (1) after labile nitrogen is released during early decomposition, much of the remaining nitrogen may be recalcitrant and does not readily release; (2) litter nitrogen may have been transformed into recalcitrant forms during microorganism metabolism^31^. This may imply grazing induced soil nitrogen losses may not be replenished immediately in the short term by the increased deposition of senesced roots. The increase in soil C:N ratio in MMM indicates that N limitation could limit plant productivity and thus C inputs to soil, or that the soil heterotrophs would be N limited with potential consequences for humification rates. The low grazing disturbance of RHM and RMH led to less nitrogen losses via leaching or NH_3_ volatilization (maintained high soil organic nitrogen level) and relatively lower root litter deposition, causing unchanged soil C:N ratio. Regression analysis between soil nitrogen change and stocking rate showed that nitrogen was retained best at a stocking rate of ~4 SE/ha (Fig. 8b), which decoupled from the optimal carbon input stocking rate of ~5 SE/ha. A stocking rate of ~5 SE/ha seems to be acceptable because soil nitrogen is still increasing, although there is potential for soil nitrogen to be a limiting in the long term.

Changes in microbial community structure have been associated with the soil carbon sequestration potential because of their substantially different biochemistries and subsequent effects on carbon stabilization^14^. Grazing regimes exerted a significant influence on soil microbial communities in our study (Fig. 7a,b), with higher F:B for RHM and RMH than HHH, HHM and MMM. The regression analysis showed that the F:B decreased with increasing stocking rate, which is consistent with the results of Grayston *et al*.^32^ who found a reduction in F:B with more intensive grazing. The changes in microbial community could be due to the replacement of the dominant grasses by annual forbs that changed the patterns of root-derived exudation, the quality of plant litter input, dung/urine deposition and other modifications to the soil physicochemical environment^33^. The fungal:bacterial dominance of a site has been associated with that site’s C-sequestration potential, with a greater potential associated with greater fungal:bacterial dominance because of higher carbon utilization efficiency of fungi^14^. However, we found that higher F:B doesn’t necessarily lead to higher carbon sequestration. For example, MMM accumulated more soil carbon than did deferred grazing regimes irrespective of its lower F:B. This may indicate that accumulation of soil carbon requires a positive imbalance between inputs to and outputs from soil and carbon accrual can be driven by an increase in photosynthetically derived carbon inputs.

In summary, grazing regimes had strong effects on plant above- and below-ground biomass production, regulating the soil carbon input in this temperate grassland. The higher proportion of grasses, plant production and soil N content observed in the deferred regimes suggested that avoiding grazing in the early growth period can both effectively maintain the local dominant species and retain soil nitrogen. However, optimal soil carbon accumulation was found with moderate grazing, which had higher belowground biomass, production and turnover, which emphasizes on the importance of roots for soil carbon input in grazing systems. The reduction in grazing pressure in the last growth stage, where growth was minimal due to an abrupt fall in temperature and precipitation, had little positive effect on the vegetation recovery, soil carbon and nitrogen accumulation.

Grazing accelerated soil nitrogen recycling but also increased nitrogen losses. Grazing induced soil nitrogen losses may not be replenished immediately in the short-term by the increased root litter deposition, which implies a potential nitrogen limitation for organic carbon formation under moderate grazing. The fungal:bacterial dominance of a site has previously been associated with that site’s C-sequestration potential, but the shifts in fungal:bacterial dominance in our study did not support this general expectation. This implies that the hypothesized positive relationship between fungal dominance and C-sequestration may not be a general phenomenon. Adjusting stocking rate to ~5SE/ha with ~40% UR can lead to the highest root production, unchanged aboveground production and elevated soil nitrogen, and give the most carbon accrual in this region. However, since our study design only resulted in five “clusters” of stocking rates (the average stocking rate of each grazing regimes) there was a limited sample size to determine the trend and the optimal position was somewhat ambiguous. However, despite this limitation, our analyses revealed informative patterns that reflect the importance of setting a grazing pressure that is appropriate to the productivity of the site. It also provides evidence that grazing management can be designed to mitigate greenhouse gases and store soil carbon.

## Methods

### Study site

This study was conducted at a temperate steppe site in Guyuan County, Hebei Province, China (41°44’N, 115°40’E), at an altitude of 1475 m. This area is a typical temperate zone characterized by a mean annual (1982–2009) precipitation of 430 mm and a mean annual temperature of 1.4 °C. Precipitation mainly falls in the growing season (June to August), which coincides with the highest temperatures. Minimum monthly mean air temperature is −18.6 °C in January and the maximum of 21.1 °C occurs in July. The site has a calcic-orthic Aridisol soil (according to the ISSS Working Group RB, 1998), with a loamy-sand texture. The plant community is dominated by *Leymus chinensis* (Trin.) Tzvel., a C3 perennial rhizomatous grass that is widely distributed in the Eurasian steppe region.

Based on the phenology of dominant and common species, and long-term seasonal patterns of temperature and precipitation in this area, we divided the growth-season (from June to end of September) into three distinct stages. Stage 1 (S1): Starting growth period (beginning of June to mid-July) when plants begin to grow. Stage 2 (S2): Active growth period (mid-July to end of August), up to when plants have finished flowering and have set seed. Stage 3 (S3): Nil growth period (end of August to end of September) when plant growth terminates because of an abrupt decrease in temperature and precipitation. We selected the study area in 2009 for the study of different grazing options on livestock production and grassland sustainability, which had been fenced to exclude grazing since 2004 because of grassland degradation. In this paper we concentrate on the effects of different grazing regimes on soil carbon. Five grazing treatments were implemented: HHH: high grazing pressure at stages 1–3; HHM-high grazing pressure in stages 1 and 2, and moderate grazing pressure in stage 3; MMM: moderate grazing pressure in stages 1–3; RMH: deferred grazing regime, with rest (stage 1), and moderate (stage 2) and high (stage 3) grazing pressure; RHM: deferred grazing regime with rest (stage 1), high (stage 2) and moderate (stage 3) grazing pressure. The detailed information of the grazing practices was shown in Supplementary Fig. S2. There was no grazing in the non-growing season including winter (from December to February) and early spring (March to May). A total of 15 plots were laid out in a completely randomized pattern; each plot was 1.5 ha in size and surrounded by wire fence. All sheep in the trial were allowed to graze all day without supplementation during the grazing period, and sheepfold was established in each plot for resting.

### Soil sampling and analyses

Soil samples were collected in mid-May 2009 (just before the experiment began) and mid-August 2013 (when aboveground biomass is at its peak) at 0–10, 10–20, 20–30 cm soil depth increments. Cores were collected using a 4-cm diameter soil corer at nine locations, with three cores bulked at each location, within each of the 15 plots. Samples from each plot were then sieved at 2-mm to remove roots. To determined soil organic carbon, the soil inorganic C (including carbonates) was removed by treating the soil with dilute hydrochloric acid (~1 M,6~7 g oven-dried soil/50 ml HCl) and stirring until no further effervescence was observed. Then the soil samples were dried at 40℃ for 4 days, ground and analyzed for carbon using an autoanalyzer (TOC, Elementar, Germany). The total nitrogen concentration was determined according to the Kjeldahl wet digestion procedure, using a 2300 Kjeltec Analyzer Unit (FOSS, Sweden).

The remainder of the fresh soil (depth 0–10 cm) was immediately stored at −80 °C and then analyzed for soil microbial communities. The microbial community in soil samples was assessed using phospholipid fatty acids (PLFAs). PLFAs were extracted from the soil as described by Bossio *et al*.^34^. The resultant fatty acid methyl esters were separated, quantified and identified using capillary gas chromatography (GC). Qualitative and quantitative fatty acid analyses were performed with an Agilent 6890 gas chromatograph (Agilent Technologies, Palo Alto, CA, USA) and the MIDI Sherlock Microbial Identification System (MIDI Inc., Newark, DE, USA).

Net nitrogen mineralization rates were determined using *in situ* soil core incubation method^35^. For each plot, nine 5 m × 5 m sampling sub-plots were established along three 40 m transect- lines. Within each sub-plot, two sharp-edged PVC tubes (5.4 cm in diameter and 15 cm in length) were driven 10 cm into the soil after the plants and litter were clipped and removed. One PVC core was sealed by a Parafilm membrane to prevent water penetration and allow gas exchange, and then left in the field for the duration of 40 days (from 15 July to 25 August). The other core was taken back to the laboratory to measure the initial inorganic N content. Soil inorganic nitrogen was extracted from a subsample of 10 g using 50 mL of 2 mol/L KCl, and N concentrations (NH_4_^+^-N and NO_3_^−^-N) were determined using a Flow-Solution analyzer (Flowsys, Ecotech, Germany). Daily net nitrogen mineralization rates were calculated by subtracting initial core total inorganic nitrogen values from incubation tube total inorganic nitrogen values, and then dividing by the incubation period.

### Determination of plant production

In mid-August 2013, nine sampling quadrats measuring 50 cm × 50 cm (three transect lines for each plot) were cut to ground level using scissors for determining aboveground biomass. The fresh samples were returned to the laboratory separated according to plant species, dried at 65 °C for 48 h and weighed. Plants were grouped into three functional types: grasses, sedges and forbs. The fresh root and foliage samples were analyzed at the China Agricultural University Laboratory for total carbon and nitrogen, and the percentage of lignin. Aboveground production was measured using 1-m^2^ movable exclosures following McNaughton *et al*.^36^, at monthly intervals between grazing periods (five cages for each plot). The UR at each grazing stage for each year was calculated by dividing the herbage mass removed by animals in a stage with the available herbage mass during this stage. Root production was measured by using in-growth soil cores^37^. Briefly, soil cores with 8 cm diameter (twelve for each plot) were extracted at the end of May at an angle of 45° to a depth of 70 cm, and roots were sieved and removed from the soil samples. A polyester mesh bag (1 mm mesh) was shaped to fit into the core hole, and the root-free soil was returned to the hole it had been collected from. After two months the soils were harvested for roots and another set of mesh bags was refilled with the sieved soil for the next period of the growth-season. The new in-growth roots were sieved, dried and weighed. Seasonal standing root biomass was sampled with a soil tuber auger 8.0 cm in diameter on six dates (May 28, June 22, July 19, August 2, August 17, and September 26) at a depth of 0–50 cm, except on August 2 when the root was sampled from soil depths of 0–10, 10–20, 20–50 cm. Annual root turnover was estimated by dividing the cumulative root production with average live root biomass obtained on each sampling date.

### Data analysis

Statistical analyses were performed using SAS Version 8.2 (SAS Institute, Cary, North Carolina, USA). ANOVA with Duncan’s multiple-range tests were used to compare the effect of grazing regimes on the plant above- and belowground production and quality data, PLFAS, soil inorganic nitrogen (NH_4_^+^ and NO_3_^−^) content and net nitrogen mineralization rate. The data for standing root biomass were run as a repeated-measures mixed model analysis. Treatment, sampling date and their interaction were considered fixed effects, and plot considered a random effect. Date was treated as a repeated measure variable. The soil organic carbon and total nitrogen data were also analyzed with the mixed procedure with treatment, soil depth and their interaction in the model as fixed effect, and plot as a random effect. Depth was treated as a repeated measure variable. Various types of variance–covariance matrices were fitted and the one with the lowest AIC value was used for the final analysis. Regress models employing the averaged stocking rate across years for each treatment as a independent variable were used to determine the relationship between stocking rate and vegetation UR (the average value for all years), vegetation production and soil net nitrogen mineralization rate. The relationships between UR and grasses, forbs proportions were also examined. We calculated the soil organic carbon and total nitrogen changes (using g kg^−1^ as unit) in top 10 cm layer by subtracting the initial soil organic carbon and total nitrogen contents from the soils collected in 2013. Relationships between stocking rates (independent variable) and top soil carbon, nitrogen change or soil Fungi: Bacteria ratios (dependent variables) were then fitted using a regression model separately. No transformation was conducted if data meet the assumption of normality, otherwise data was log-transformed prior to analysis to ensure normality.

## Additional Information

**How to cite this article**: Chen, W. *et al*. Improved grazing management may increase soil carbon sequestration in temperate steppe. *Sci. Rep*. **5**, 10892; doi: 10.1038/srep10892 (2015).

## Supplementary Material

Supplementary Information
