# Supplementary material for: Improved grazing management may increase soil carbon sequestration in temperate steppe
Source: Sci Rep. 2015 Jul 3;5:10892. doi: 10.1038/srep10892 (PMC4490272; doi:10.1038/srep10892)
Supplement: Supplementary Information [file srep10892-s1.pdf]

Improved grazing management may increase soil carbon sequestration in temperate steppe

Wenqing Chen[1], Ding Huang[1], Nan Liu[1], Yingjun Zhang[1,*], Warwick B. Badgery[2], Xiaoya Wang[1] & Yue Shen[1]

[1] Department of Grassland Science, College of Animal Science and Technology, China Agricultural University, West Road 2 Yuan Ming Yuan, Beijing 100193, P.R. China, [2] New South Wales Department of Primary Industries, Orange Agricultural Institute, Orange, NSW 2800, Australia

*Corresponding author: Tel: +86 010 62733380.

E-mail address: zhangyj@cau.edu.cn

Supplemental Figure S1. Experimental site, relative position and plot layout. The map was created using software ArcGIS 10.2 (Esri, RedLands, California, USA).

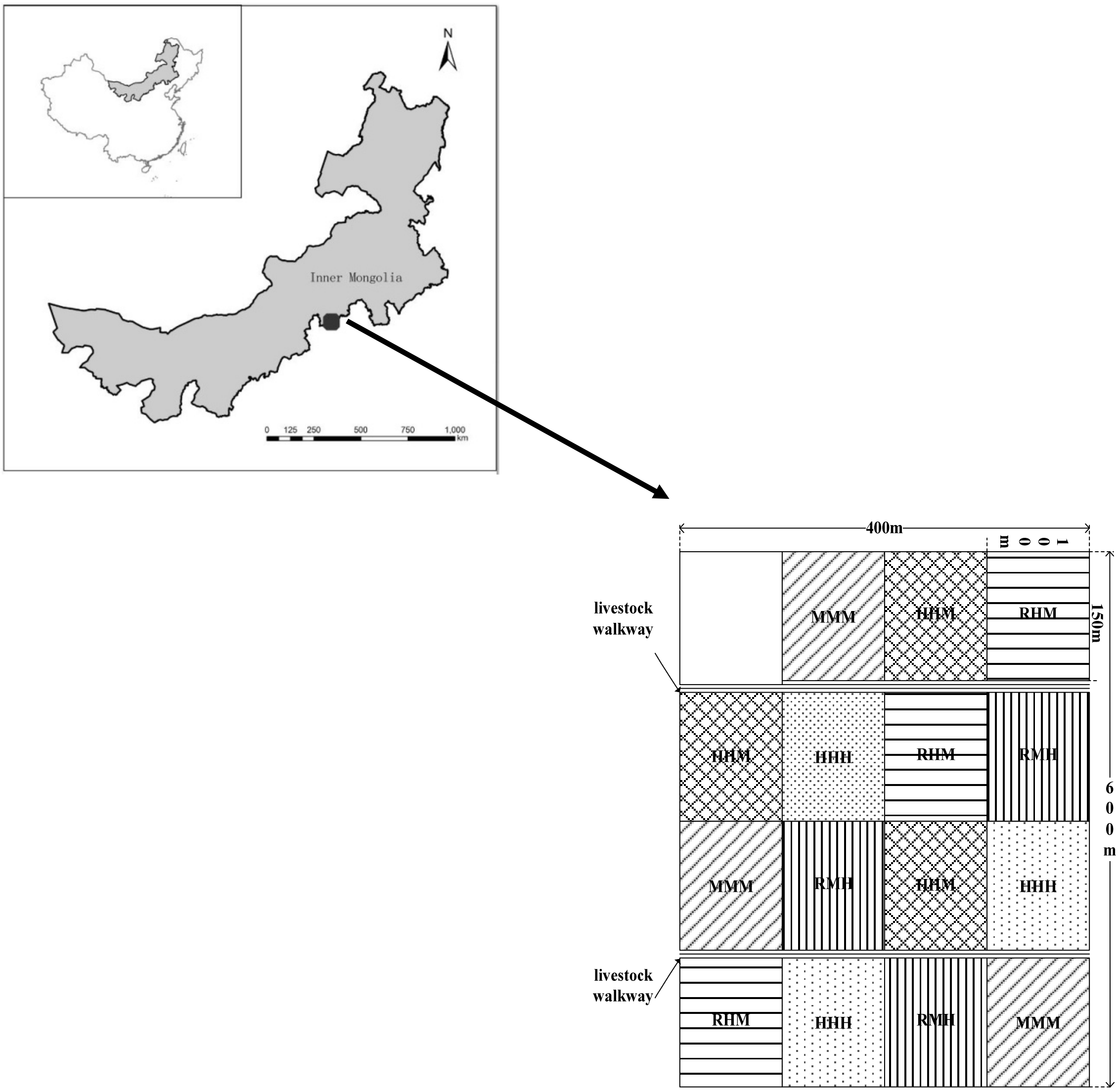

Supplemental Figure S2. Averaged stocking rates (SE/ha; 1 sheep equivalent (SE) = a 50kg reference weight animal to adjust for different size

animals over time) across years in different stages (a) and averaged stocking rates across years and stages (b) for each regime.

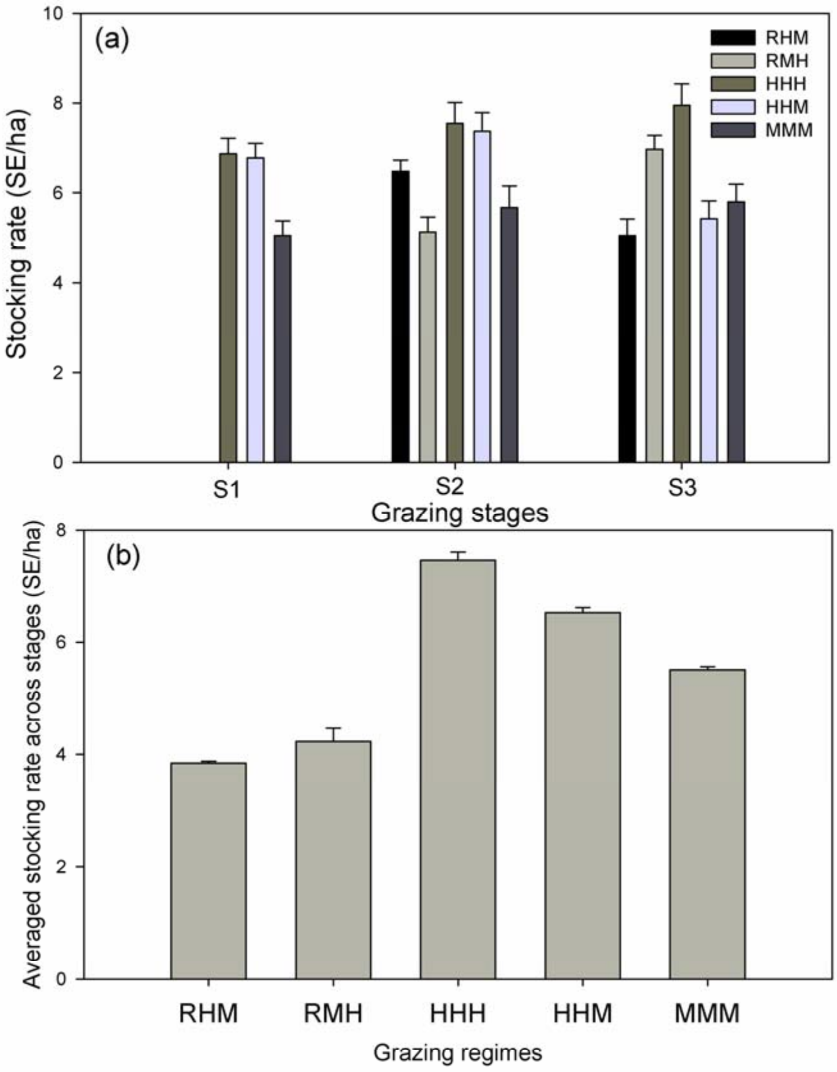

Supplemental Figure S3. Averaged vegetation utilization rates (UR) across years in different stages (a) and averaged utilization rate across years and stages (b) for each regime.

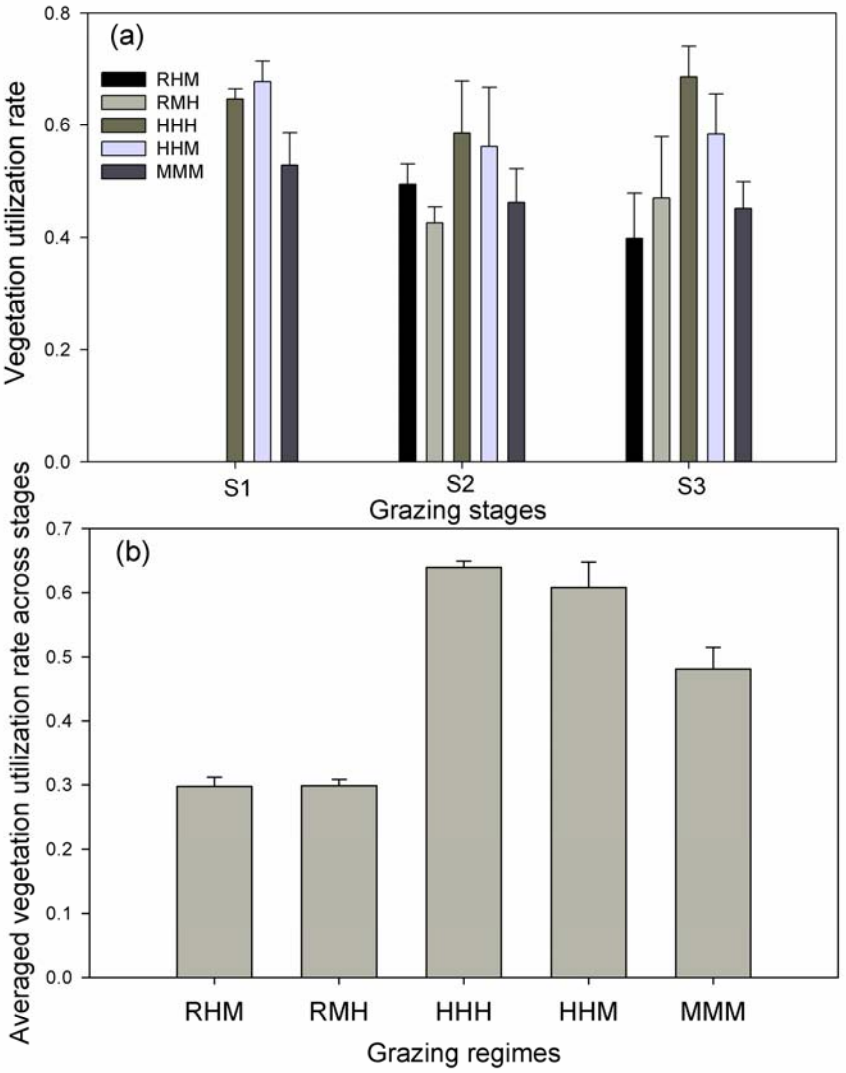

Supplemental Figure S4. Response of N content, lignin content, lignin:N ratio and C:N ratio of tissue in shoots (a) and roots (b) following different grazing regimes. Bar groups with different lowercase letters indicate significant differences ($P < 0.05$) between regimes. Values are mean±SE.

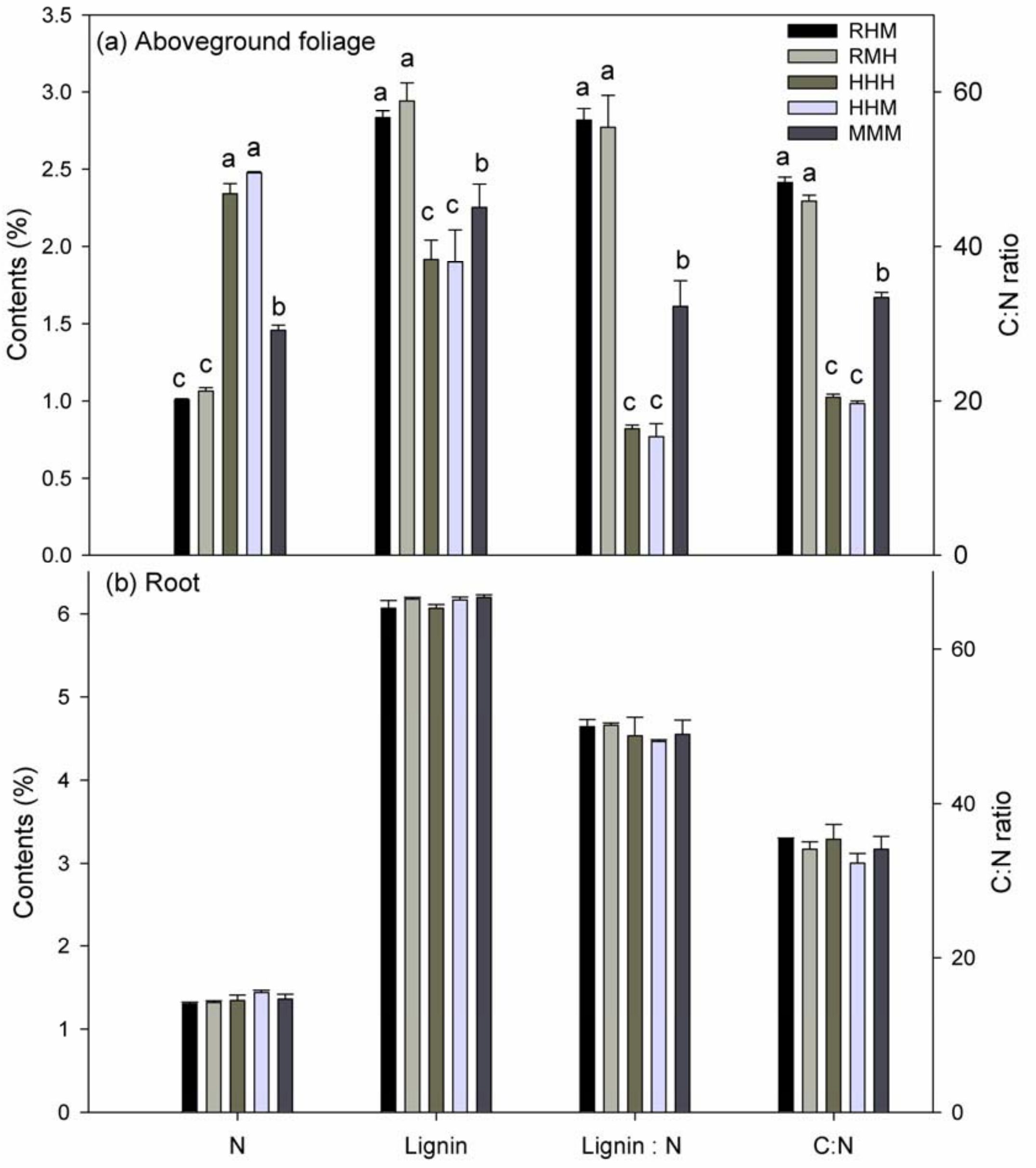

(a) Aboveground foliage
(b) Root
RHM
RMH
HHH
HHM
MMM
Contents (%)
C:N ratio
N
Lignin
Lignin : N
C:N
